# Case Report: Presumed ocular tuberculosis masquerading as acute retinal necrosis: the diagnostic and therapeutic journey of an elderly female

**DOI:** 10.3389/fmed.2026.1900711

**Published:** 2026-07-31

**Authors:** Cheng Zuo, Ya-Li Yang, Jun-Feng Yang

**Affiliations:** Department of Ophthalmology, The Third People's Hospital of Chengdu, The Affiliated Hospital of Southwest Jiao Tong University, Chengdu, Sichuan, China

**Keywords:** acute retinal necrosis (ARN), anti-tuberculosis therapy (ATT), masquerade, ocular tuberculosis, presumed ocular tuberculosis

## Abstract

**Background:**

Acute retinal necrosis (ARN) is typically caused by varicella-zoster or herpes simplex virus, but Mycobacterium tuberculosis can present an identical clinical picture. Distinguishing tuberculous uveitis (TBU) from ARN is challenging when etiological tests are unavailable.

**Case presentation:**

A 78-year-old woman presented with rapid visual loss (hand motion) in the left eye. Examination revealed elevated intraocular pressure, mutton-fat keratic precipitates, severe anterior chamber and vitreous inflammation, retinal arteritis, and peripheral necrotic lesions, which met the 2015 Japanese diagnostic criteria for presumed ARN with undetermined virology. Standard antiviral therapy (famciclovir, two intravitreal ganciclovir injections, and corticosteroids) failed to halt the progression. An interferon-gamma release assay (IGRA) was positive, and chest CT showed non-specific changes. After discontinuing antivirals, diagnostic anti-tuberculosis therapy (ATT) led to rapid improvement: visual acuity reached 0.1, inflammation resolved, and necrotic lesions became pigmented scars. Over a 12-month follow-up period, which included 6 months of observation after completion of ATT, the patient maintained a stable BCVA of 0.1, with no evidence of retinal detachment or disease recurrence. The final diagnosis was revised to presumed ocular tuberculosis.

**Conclusion:**

Presumed ocular tuberculosis can clinically mimic ARN. When suspected ARN progresses despite adequate antiviral treatment, intraocular tuberculosis should be included in the differential diagnosis. When a definitive diagnosis cannot be established, an ATT trial guided by indirect evidence such as a positive IGRA is a reasonable clinical option. It should be clarified that this trial only supports a presumptive diagnosis rather than a definitive diagnosis.

## Introduction

Acute retinal necrosis syndrome (ARN) is a rapidly progressive, blinding necrotizing retinitis, most commonly caused by the varicella-zoster virus (VZV) or herpes simplex virus (HSV). The diagnosis is primarily based on the classic clinical triad, which consists of peripheral retinal necrosis, retinal arteritis, and inflammation of the vitreous and anterior chamber. However, many other pathogens, including cytomegalovirus, Toxoplasma gondii, and Mycobacterium tuberculosis, can also produce ARN-like presentations. Ocular tuberculosis (TBU) is a classic masquerade syndrome that mimics many forms of uveitis, including ARN, leading to diagnostic delays and inappropriate treatment (1). Since specific tests (e.g., polymerase chain reaction [PCR] on intraocular fluid) are often unavailable in real-world practice, clinicians frequently rely on a combination of clinical judgment, indirect laboratory markers, and therapeutic response.

The 2015 Japanese diagnostic criteria for ARN categorize diagnoses into two levels (2): (1) “virus-confirmed ARN” requires early-stage ocular manifestations (anterior chamber cells or mutton-fat KPs, plus peripheral yellow-white retinal lesions), at least one of five clinical features (rapid circumferential progression, retinal breaks/detachment, vascular occlusion, optic atrophy, or response to antiviral treatment), and a positive HSV/VZV result from intraocular fluid PCR; (2) “virus-unconfirmed ARN” requires four out of six early-stage findings (including items 1a and 1b), any two of the five clinical features, and either a negative PCR result or an unperformed PCR test. The patient in this report met the criteria for virus-unconfirmed ARN: she had four early-stage findings and two clinical features, and no virologic testing was carried out.

Here, we report a case that initially met all the clinical criteria for ARN. It did not respond to standard antiviral therapy but improved significantly with diagnostic anti-tuberculosis therapy (ATT). This case demonstrates that an ARN-like condition can be caused by tuberculosis in the absence of any systemic tuberculosis symptoms. It also presents a clear decision-making pathway that uses treatment failure as a trigger to re-evaluate the diagnosis and the successful application of therapeutic diagnosis when etiological confirmation is not possible. We aim to increase clinical awareness of ocular tuberculosis masquerading as ARN and propose a practical algorithm for managing similar challenging cases.

## Case presentation

A 78-year-old female presented with progressive visual deterioration in the left eye for 1 week. She reported no ocular redness, pain, or other irritative symptoms. Her medical history was notable for hypertension, which was well controlled. There was no documented history of tuberculosis (TB), known contact with TB patients, or prior use of corticosteroids or immunosuppressive drugs.

**Figure 1:**
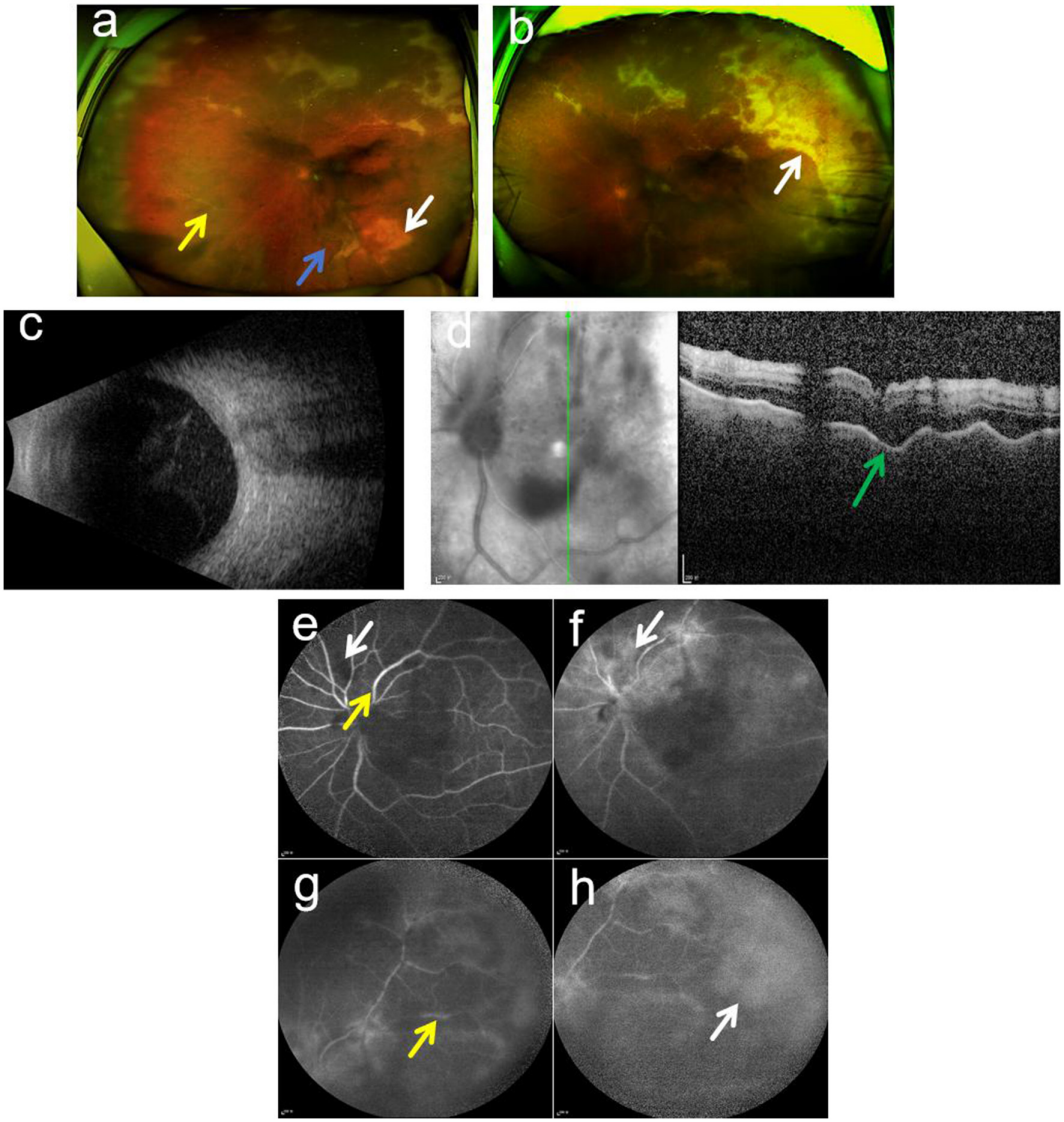
Multimodal imaging of the left eye at initial presentation. **(a, b)** Color fundus photograph depicting flocculent opacity in the vitreous and an exudative-appearing floater (blue arrow). The optic disc is hyperemic with slightly blurred margins. Segmental retinal vessel occlusion is evident as white lines (yellow arrow). Numerous patchy yellow-white necrotic lesions are observed in the mid-peripheral and peripheral retina, with confluent lesions in the superior temporal quadrant (white arrow). **(c)** B-scan ultrasonography demonstrating dense punctate and flocculent opacities within the vitreous cavity. **(d)** OCT image showing disorganized inner retinal architecture, increased retinal thickness, and undulating elevation of the RPE layer in the macular region (green arrow). **(e–h)** FFA: **(e)** The early phase shows segmental hypofluorescence of retinal vessel walls (yellow arrow) and hypofluorescence corresponding to the necrotic lesions (white arrow). **(f, g)** Mid-phase images showing hyperfluorescence of the necrotic lesions (white arrow) and pronounced vascular leakage (yellow arrow). **(h)** Late phase image showing fluorescein leakage from the necrotic lesions (white arrow), accompanied by diffuse retinal hyperfluorescence.

Initial ophthalmic examination revealed that the best-corrected visual acuity (BCVA) was 0.4 in the right eye and hand motion (HM) at 30 cm in the left eye. The intraocular pressure (IOP) was 15 mmHg in the right eye and 38 mmHg in the left eye. Slit-lamp examination of the anterior segment revealed no obvious abnormalities in the right eye except for lens opacity. In the left eye, the cornea was hazy and edematous, with several mutton-fat keratic precipitates (KPs) located inferiorly on the corneal endothelium. Anterior chamber cells were graded 3+ and flare3+. The lens was opaque. A dilated fundus examination of the posterior segment was unremarked in the right eye. In the left eye, the vitreous presented flocculent opacity with severe cellular infiltration (3+), and an exudative-appearing floater was observed inferiorly. The optic disc was hyperemic with slightly blurred margins. Retinal edema was noted, along with segmental occlusion of retinal arterioles and venules, appearing as white lines. Numerous patchy yellow-white necrotic lesions were observed in the mid-peripheral and peripheral retina, with confluent lesions in the superior temporal quadrant (Figures 1a, b).

To further evaluate the diagnosis, multimodal imaging was performed. Ocular B-scan ultrasonography revealed dense punctate and flocculent opacities in the vitreous cavity of the left eye, with a positive posterior movement test (Figure 1c). Optical coherence tomography (OCT) showed disorganized inner retinal architecture, increased retinal thickness, and undulating elevation of the retinal pigment epithelium (RPE) layer in the macular region (Figure 1d). Fundus fluorescein angiography (FFA) demonstrated segmental hyperfluorescence of the retinal vessel walls in the early phase and hypofluorescence corresponding to necrotic lesions. In the late phase, the necrotic lesions showed hyperfluorescence, accompanied by diffuse retinal hyperfluorescence and pronounced vascular leakage (Figures 1e–h).

Based on the clinical presentation and imaging findings, the patient met the criteria for presumed ARN with undetermined virology according to the 2015 Japanese diagnostic criteria for ARN (2). A preliminary diagnosis of ARN was established.

After admission, a comprehensive work-up was conducted, including complete blood count, erythrocyte sedimentation rate, liver and renal function tests, systemic infection panel, autoimmune panel, TORCH panel, interferon gamma release assay (IGRA), and chest computed tomography (CT). Meanwhile, immediate treatment was initiated. Topical medications included prednisolone acetate ophthalmic solution, atropine ophthalmic gel, and carteolol hydrochloride ophthalmic solution. An intravitreal injection of ganciclovir (2 mg/0.1 ml) was administered. Aqueous humor sampling was proposed but declined by the patient. Systemic therapy comprised oral famciclovir (500 mg three times daily), aspirin (100 mg once daily), and mecobalamin (0.5 mg three times daily). Oral prednisone (30 mg once daily) was added 2 days after treatment initiation.

**Figure 2:**
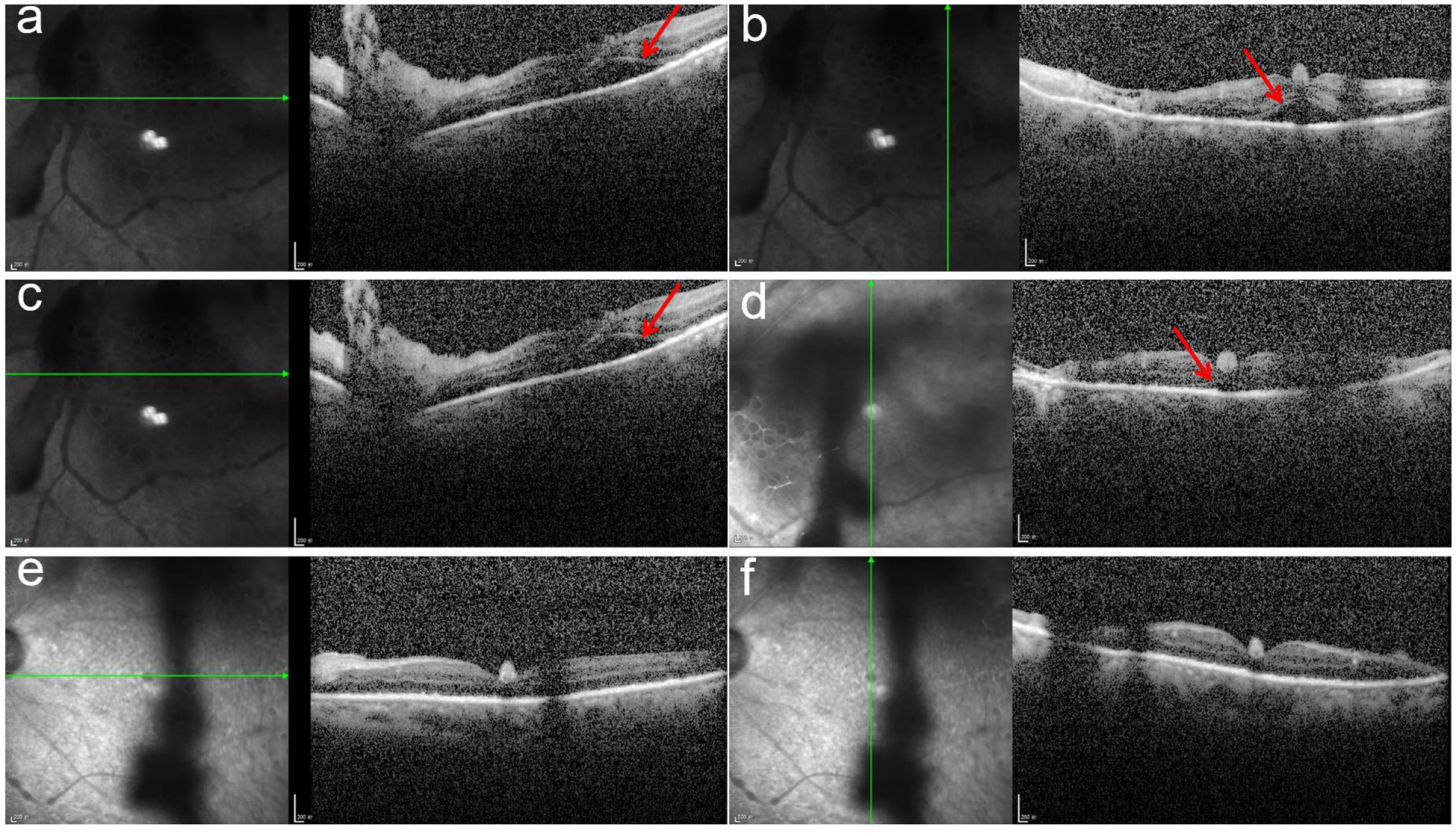
Follow-up OCT images of the left eye. **(a, b)** 3 days after the second intravitreal ganciclovir injection: OCT shows neurosensory detachment at the fovea (red arrow). **(c, d)** 7 days after initiation of ATT: OCT shows partial resolution of the macular neurosensory detachment (red arrow). (**e, f**) 19 days after ATT, OCT reveals complete reattachment of the macular neurosensory retina.

6 days after starting antiviral therapy, the visual acuity of the left eye improved to counting fingers (CF), with partial resolution of anterior chamber and vitreous inflammation. However, new yellow-white retinal necrotic lesions developed along the inferotemporal vascular arcade. A second intravitreal ganciclovir injection (2 mg/0.1 ml) was promptly administered. 3 days after the second injection, the patient's condition deteriorated abruptly. The inferotemporal necrotic lesions enlarged with patchy hemorrhage. More retinal vessels appeared as white lines, and IOP rebounded to 25 mmHg. OCT revealed neurosensory detachment at the fovea (Figures 2a, b). Despite standard antiviral and corticosteroid combination therapy, the disease continued to progress.

At this juncture, laboratory results became available. IGRA was positive. Chest CT showed inflammatory changes, ground-glass nodules, and bilateral pleural thickening. TORCH testing was positive for IgG but negative for IgM against Toxoplasma gondii, rubella virus, cytomegalovirus, and herpes simplex virus. Systemic infection and autoimmune panels were unremarkable. A consultation with the department of respiratory medicine was obtained. The consensus was that chest CT indicated inflammatory changes without definitive evidence of active pulmonary tuberculosis. However, considering the poor response to antiviral therapy and a positive IGRA, a diagnostic trial of anti-tuberculosis therapy (ATT) was recommended, despite the atypical features. After obtaining thorough informed consent, antiviral medications were discontinued on the 12th day of treatment, and ATT was initiated: isoniazid 0.3 g once daily, pyrazinamide 0.5 g twice daily, and rifapentine 0.45 g twice weekly.

This treatment regimen was developed by the Department of Infectious Diseases following multidisciplinary consultation. Ethambutol was intentionally omitted for this patient for two key reasons: the patient is 78 years old, and the drug carries a potential risk of irreversible optic neuropathy that could further damage her already impaired vision. Rifapentine was selected over daily rifampicin to improve treatment adherence for this elderly patient. Although this regimen differs from the standard daily four-drug RIPE regimen, it is an individualized, risk-benefit balanced approach administered under close monitoring. Topical corticosteroid and IOP-lowering agents were continued, but no further intravitreal injections were performed.

Seven days after initiating ATT, BCVA in the left eye improved to 0.02, and IOP normalized. Anterior chamber inflammation resolved, vitreous opacities decreased significantly, and cellular infiltration was reduced. Retinal necrotic lesions diminished in size with sharper margins. OCT showed partial resolution of the macular neurosensory detachment (Figures 2c, d). 19 days after ATT, BCVA further improved to 0.1. Most retinal necrotic lesions had been replaced by pigmented scars, and no new active lesions were observed. OCT demonstrated complete reattachment of the macular neurosensory retina. Although mild disorganization of retinal lamination persisted, the morphology was markedly improved compared with baseline (Figures 2e, f). The final diagnosis was revised to presumed ocular tuberculosis of the left eye.

The patient completed the full six-month course of ATT, which included a 2-month intensive phase followed by a 4-month continuation phase. 1 week after starting ATT treatment, the daily oral prednisone dosage was gradually tapered from 30 mg, with a 5 mg reduction each week, and the drug was fully discontinued within 2 months. Across the treatment period and an additional 6 months of follow-up, bringing the total period to 12 months, ocular inflammation remained completely quiescent during and after corticosteroid tapering, with no signs of recurrence. No rhegmatogenous retinal detachment or new necrotic lesions developed, and the patient's left-eye BCVA remained stable at 0.1 at the final follow-up visit.

**Figure 3:**
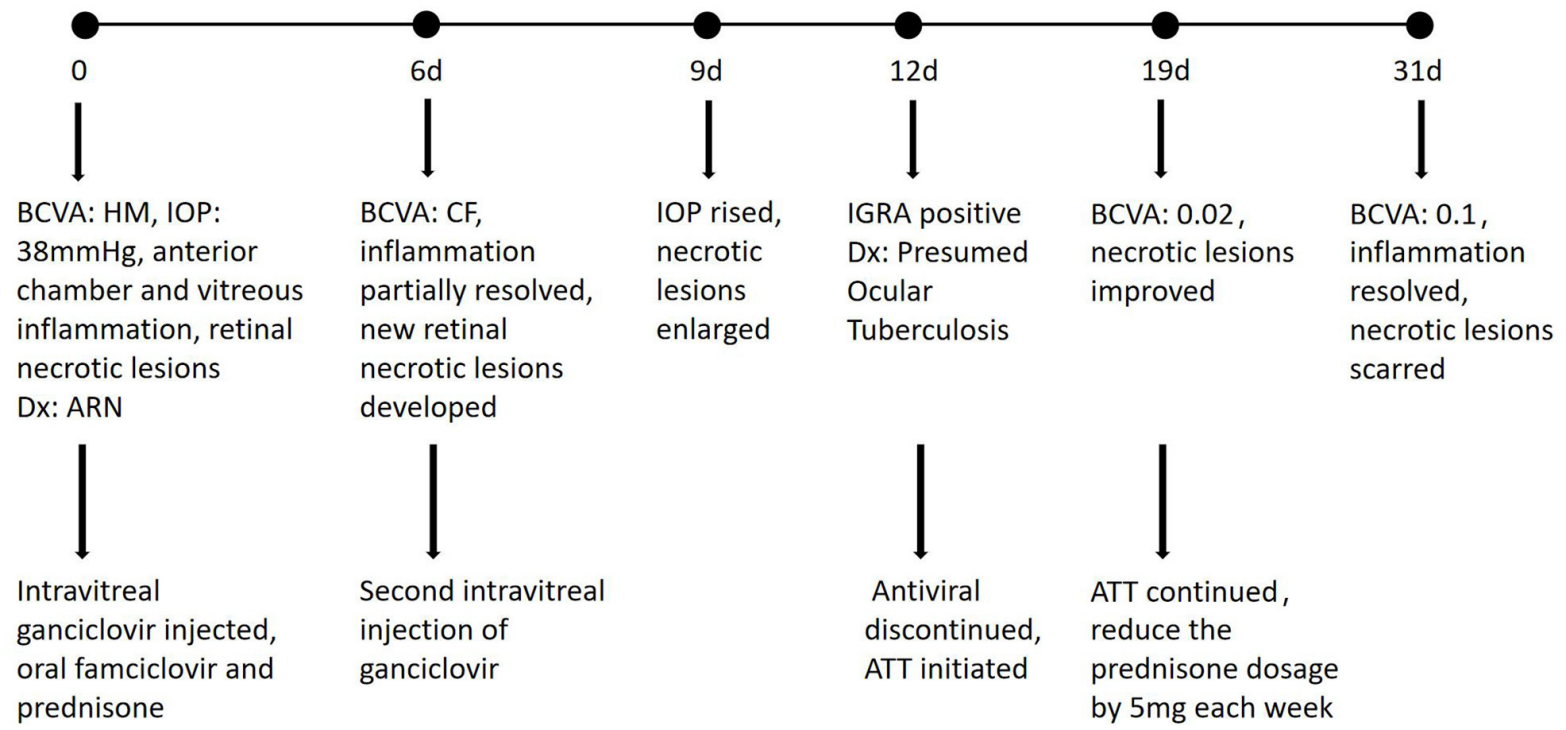
Clinical timeline. The horizontal axis shows time. Key events include: onset of ocular symptoms, preliminary diagnosis of ARN and initiation of antiviral treatment (0 days), second intravitreal injection (6 days), worsening of fundus lesions (9 days), diagnosis of TBU and initiation of ATT (12 days), clinical improvement at 19 days and 31 days. ATT, anti-tuberculosis therapy; Dx, diagnosis; TBU, tuberculous uveitis; IOP: intraocular pressure; BCVA, best-corrected visual acuity.

The key events from diagnosis through recovery are summarized in the clinical timeline presented in Figure 3.

## Discussion

ARN and ocular tuberculosis are two types of uveitis with distinctly different etiologies but highly overlapping clinical presentations. ARN is primarily caused by herpesvirus infection, and its pathogenesis involves the direct cytopathic effect of the virus on the neurosensory retina. In contrast, ocular tuberculosis is caused by Mycobacterium tuberculosis, and its pathogenesis may involve either direct infection by the pathogen or a severe immune-inflammatory response triggered by mycobacterial antigens. Both conditions can present with symptoms such as ocular redness, pain, floaters, and decreased vision. Clinically, both may exhibit anterior chamber inflammation, vitreous opacities, retinal vasculitis, and necrotizing lesions (3, 4). This overlap in clinical phenotypes, combined with the difficulty in obtaining etiological evidence, makes differentiation between the two a common challenge in ophthalmic practice. The unique aspect of this case lies in the fact that the patient's clinical presentation was highly consistent with the diagnostic criteria for ARN established by the Japanese ARN Study Group in 2015 (2), yet the underlying etiology ultimately pointed to tuberculosis. This phenomenon indicates that the range of pathogens that can cause ARN as a clinical syndrome might be wider than traditionally thought. Clinicians should recognize that a typical ARN-like presentation is not limited to herpesviruses; presumed ocular tuberculosis can also imitate it.

Beyond herpesviruses and Mycobacterium tuberculosis, other pathogens can also cause necrotizing retinitis. In immunocompetent hosts, cytomegalovirus (CMV) retinitis typically presents with hemorrhagic lesions and follows a more indolent clinical course. Toxoplasmic retinochoroiditis is characterized by a prominent overlying vitreous reaction, and generally does not cause retinal arteritis of the same severity. While syphilitic retinitis can also mimic ARN, it is usually accompanied by additional ocular manifestations such as interstitial keratitis or neuroretinitis. In the present case, negative TORCH IgM results, normal syphilis serology, and the absence of characteristic features of toxoplasmic or syphilitic infection made these alternative diagnoses far less likely. However, since no PCR testing was performed, these conditions cannot be definitively ruled out, which further underscores that our final diagnosis remains presumptive.

The 2017 American Academy of Ophthalmology expert consensus on the diagnosis and management of ARN (5) clearly states that for patients with a clinically typical presentation suggestive of ARN, antiviral therapy may be initiated without waiting for laboratory confirmation. The initial treatment strategy in this case strictly adhered to this principle, employing systemic and local antiviral therapy, followed by adjunctive corticosteroids 48–72 h later (5, 6). However, despite the administration of standard and adequate antiviral therapy combined with corticosteroids, the patient's condition did not improve; instead, it exhibited paradoxical progression. This situation is rare in standard ARN and serves as a crucial hint for re-evaluating the diagnosis. This clinical experience highlights that a significant deviation in treatment response from expectations should prompt a re-evaluation of the diagnosis, rather than merely being regarded as a result of disease severity or inadequate treatment.

The ATT regimen used in this case warrants specific discussion. While standard initial-phase therapy for extrapulmonary tuberculosis is a daily four-drug regimen (consisting of isoniazid, rifampicin, pyrazinamide, and ethambutol, our patient received a three-drug regimen that substituted twice-weekly rifapentine for daily rifampicin and excluded ethambutol. This decision was made by the infectious disease specialists, taking into account the patient's advanced age, the risk of ethambutol-induced optic neuritis, and the need to support treatment adherence for this elderly patient. The rapid, dramatic clinical response to this individualized regimen confirms its effectiveness in this specific case. Nevertheless, we emphasize that this was a case-specific clinical decision and does not constitute a recommendation to deviate from standard treatment guidelines.

The gold standard for confirming both ARN and ocular tuberculosis is etiological diagnosis. However, intraocular tissue biopsy is technically demanding, involves considerable risk of injury, and even when it succeeds, it often produces low positive rates, preventing its use as a standard diagnostic procedure. While advances in laboratory techniques have led to the widespread application of intraocular fluid polymerase chain reaction (PCR) and metagenomic next-generation sequencing in clinical practice (7, 8), their implementation is often constrained in real world settings by factors such as the invasiveness of the procedure and limited hospital resources. When clinically feasible, intraocular fluid polymerase chain reaction (PCR) testing for HSV/VZV remains the preferred diagnostic approach for ARN. However, our patient declined to undergo intraocular fluid sampling. The absence of PCR results significantly compromises diagnostic certainty, meaning that other potential etiologies, including viral, toxoplasmic, and syphilitic causes, cannot be definitively ruled out.

In this case, lacking etiological evidence from the patient, an alternative diagnostic pathway was constructed. This pathway utilizes indirect evidence of failed antiviral therapy combined with a positive IGRA result as epidemiological indicators, leading to the clinical decision to initiate diagnostic ATT, which is finally confirmed by a positive treatment response. The rapid clinical improvement seen after the initiation of ATT provides strong supportive evidence for the presumptive diagnosis of ocular tuberculosis. We acknowledge, however, that this improvement does not confirm a causal relationship. The concurrent administration of corticosteroids, the two-week course of antiviral therapy the patient received prior to starting ATT, and the possibility of spontaneous disease stabilization cannot be completely ruled out. Even so, the close temporal association between initiating ATT and the onset of clinical improvement, with the patient's condition deteriorating before treament and resolving rapidly afterward, makes a genuine therapeutic effect the most plausible explanation. This evidence-chain decision-making framework is particularly beneficial for situations involving older patients, where invasive methods are unsuitable, or in settings with scarce resources. This observation aligns with the findings of Chong et al., where the intraocular fluid PCR could be negative, and the treatment response itself served as the basis for the final diagnosis (9).

For ocular tuberculosis, even in the absence of systemic active disease, systemic ATT remains the cornerstone of treatment when ocular findings and ancillary test results support a presumptive diagnosis (10). Even if there are no systemic signs, antituberculosis treatment should be considered when ocular findings strongly suggest tuberculosis and other potential causes have been ruled out. The favorable outcome in this case confirms the necessity of systemic ATT for ocular tuberculosis. Furthermore, the decision-making process in this case highlights the critical value of multidisciplinary collaboration (MDT). The collaboration with a respiratory medicine expert enabled a detailed assessment of the atypical chest CT findings and the positive IGRA result, resulting in a well-founded decision on diagnostic therapy while managing clinical risk.

Based on our experience with this case, we propose a practical decision-making pathway for clinical reference: for patients with suspected ARN whose condition continues to progress despite standard antiviral therapy, intraocular tuberculosis should be included in the differential diagnosis. Immunological tests such as IGRA are of significant value in screening for uveitis of unknown origin. In the absence of etiological evidence, a decision-making pathway following the paradigm of ‘therapeutic paradox + indirect evidence + treatment response' represents a safe and effective alternative. Multidisciplinary collaboration helps improve diagnostic accuracy and mitigate clinical risks.

### Limitations

We acknowledge several important limitations. First, the most critical limitation of this report is the lack of etiological evidence obtained from intraocular fluid, specifically our PCR tests for HSV/VZV and Mycobacterium tuberculosis returned negative results. Without this evidence, the diagnosis of ocular tuberculosis and the exclusion of viral ARN and other causes of necrotizing retinitis remain presumptive. For patients with suspected ARN, HSV/VZV PCR testing should be performed whenever clinically feasible. Thus, the causal link to Mycobacterium tuberculosis remains inferential rather than definitively proven. While the observed therapeutic response is compelling, it can still be attributed to alternative explanations, including the delayed effects of prior treatments and spontaneous disease stabilization. From the perspective of evidence-based medicine, obtaining microbiological or molecular confirmation should always be the priority when clinically possible. Second, there was room for improvement in our clinical decision-making process. If we had adjusted the treatment strategy more promptly when we first observed antiviral failure, we could have halted disease progression earlier and potentially achieved a better final visual prognosis. Third, the ATT regimen we used was atypical and individualized, so the generalizability of this approach for other patients with ocular tuberculosis remains uncertain. Fourth, as this is only a single case report, the improvement observed after ATT cannot definitively establish a causal relationship. Although we have supplemented this report with 12-month follow-up data confirming sustained disease stability, long-term clinical surveillance is still required. In addition, the patient's advanced age and the complete lack of systemic tuberculosis symptoms or risk factors represent atypical features for ocular tuberculosis, which further restricts the generalizability of our findings. Given these limitations, our conclusions are hypothesis-generating rather than definitive. Larger case series, prospective studies, and ideally controlled clinical trials are required to validate the diagnostic approach proposed in this report.

## Conclusion

Infectious uveitis remains a major cause of blindness, and the overlap between ARN and ocular tuberculosis frequently leads to diagnostic delay or misdiagnosis. This case demonstrates that presumed ocular tuberculosis can produce a clinical picture indistinguishable from ARN, including full satisfaction of the Japanese diagnostic criteria. When a suspected ARN case fails to respond to standard antiviral therapy, intraocular tuberculosis should be actively considered. In the absence of definitive etiological confirmation, a diagnostic trial of ATT, guided by positive IGRA and informed by multidisciplinary discussion, may be a vision-saving intervention. However, the diagnosis in this case remains presumptive in the absence of microbiological confirmation. The dramatic response to ATT, while highly suggestive, cannot replace the gold standard for etiological diagnosis. Clinicians should interpret this single observation cautiously. Whenever possible, efforts to obtain microbiological or molecular confirmation should be pursued. This report adds to the growing literature on tuberculous masquerade syndromes and underscores the importance of recognizing treatment failure as a diagnostic opportunity.

## Data Availability

The original contributions presented in the study are included in the article/supplementary material, further inquiries can be directed to the corresponding author.
